# Boundary cap neural crest stem cells exhibit remarkable resilience to environmental stressors associated with International Space Station mission

**DOI:** 10.1038/s41526-026-00646-5

**Published:** 2026-08-06

**Authors:** Robert Fredriksson, Yilin Han, Xiangyi Du, Craig A. Sexton, Povilas Barasa, Federica Zanotti, Nicola B. Hamilton, Keisuke Ito, Barbara Zavan, Elena N. Kozlova

**Affiliations:** 1 Department of Pharmaceutical Biosciences, Uppsala University, Uppsala, Sweden; 2 Department of Immunology, Genetics and Pathology, Uppsala University, Uppsala, Sweden; 3 Wolfson Sensory, Pain and Regeneration Centre, Institute of Psychiatry, Psychology and Neuroscience, King’s College, London, UK; 4 Institute of Biochemistry, Life Sciences Center, Vilnius University, Vilnius, Lithuania; 5 Department of Translational Medical Sciences, University of Ferrara, Ferrara, Italy; 6 Ruth L. and David S. Gottesman Institute for Stem Cell and Regenerative Medicine Research, Albert Einstein College of Medicine, Bronx, NY USA; 7 Departments of Cell Biology, Medicine and Oncology, Albert Einstein College of Medicine, Bronx, NY USA

**Keywords:** Cell biology, Developmental biology, Neuroscience, Stem cells

## Abstract

Boundary cap neural crest stem cells (BCs) previously demonstrated resilience during short-term microgravity exposure. In this study, BCs were sent to the International Space Station aboard Axiom Mission 3. Owing to launch regulations and weather delays, cells remained outside controlled culture conditions longer than anticipated, exceeding expected survival limits. Three BC populations were included: naive BCs (NBC), once-flown BCs (V15), and twice-flown BCs (V1415), cultured as neurospheres or within 3D-printed scaffolds. Corresponding ground controls were maintained under matched conditions. Only V1415 cells survived in both flight and ground groups, while NBC cells survived exclusively after spaceflight. V15 cells did not survive. All surviving cells exhibited reduced proliferation, contrasting with the enhanced proliferation reported after short-term microgravity exposure. Following 1 month of post-flight expansion, surviving cells retained the capacity to differentiate into neurons and glial cells without detectable electrophysiological impairment. Space-exposed NBC cultures showed increased glial differentiation, whereas V1415 flight cultures displayed a higher neuronal proportion than ground controls. BCs cultured in 3D-printed scaffolds exhibited robust survival and significantly increased proliferation after space exposure. Exosome analysis identified miRNAs associated with enhanced proliferation in flight samples. These findings demonstrate the remarkable stress resistance, developmental plasticity, and adaptability of BCs during prolonged spaceflight conditions.

## Introduction

Boundary cap neural crest stem cells (BCs) constitute a transient, but highly specialized population that resides at the dorsal and ventral root transitional zones during embryonic development, forming the interface between the central and peripheral nervous systems. These cells retain broad multipotency: in vitro they can differentiate into multiple neuronal and glial subtypes^1–3^, while in vivo transplantation studies have demonstrated their capacity to integrate into both the peripheral nervous system^4^ and the central nervous system^5–7^. Beyond their neural potential, BCs and BC-derived glial cells exert remarkably protective effects on vulnerable or diseased tissues, including insulin-producing β-cells^8–13^ and degenerating motor neurons^14–16^. BC-derived glial cells also display extraordinary resistance to oxidative stress and metabolic challenge^14^. These features—developmental plasticity, stress tolerance, and robust trophic support—identify BCs as an excellent model for exploring cellular responses to extreme environmental conditions.

Spaceflight represents one such extreme environment. Microgravity induces profound alterations in cytoskeletal organization^17^, cell polarity^18^, metabolism^19^, and proliferation^20^ across diverse cell types. However, spaceflight experiments also involve prolonged pre-launch handling, transport, sealed culture conditions, and delayed recovery, which may additionally influence cellular behavior. Understanding how stem cells adapt to these conditions, including microgravity, is crucial both for human space exploration and for fundamental cell biology, as these conditions unmask mechanisms of cellular plasticity that are less evident on Earth.

Previous experiments using MASER 14 and MASER 15 sounding rockets revealed that BCs withstand brief exposure to microgravity, retain their differentiation capacity, and unexpectedly exhibit increased proliferation after flight^21,22^. These short-duration missions, however, did not elucidate the limits of BC survival or the strategies they employ under long-term microgravity, nutrient restriction, and absence of regulated atmospheric and incubator conditions.

The present experiment was designed to address these knowledge gaps by sending BCs to the International Space Station (ISS) as part of the Swedish Muninn contribution to Axiom Mission 3, flown with European Space Agency (ESA) astronaut Marcus Wandt. We examined three BC populations differing in their prior exposure to microgravity—naive cells and cells previously flown once or twice on sounding rocket—to determine whether past spaceflight experience influences resilience, proliferation kinetics, and differentiation outcomes after prolonged exposure to microgravity. According to mission regulations, all samples had to be delivered to the launch site 48 h before planned departure. Unexpected weather-related delays further prolonged the total time outside controlled incubator conditions, inadvertently extending BC exposure beyond their previously defined survival limit. This created a unique opportunity to investigate how BCs respond to combined mission-associated stress conditions including microgravity, transport, sealing, and prolonged ambient exposure rather than isolated gravitational unloading.

The goals of this study were therefore fourfold: (1) to assess the survival of BCs under extended pre-launch and in-flight stress; (2) to determine whether prior microgravity exposure confers increased resilience, as a potential form of preconditioning; (3) to evaluate post-flight proliferation, differentiation, and electrophysiological function; and (4) to test whether 3D-printed scaffolds improve BC survival in combined mission-related stress conditions compared to free-floating neurospheres. Together, these analyses provide new insight into how neural crest–derived stem cells adapt to extreme environmental perturbations and identify cellular properties that may be leveraged for future regenerative medicine applications in space and on Earth.

## Results

### General observations

**Fig. 1:**
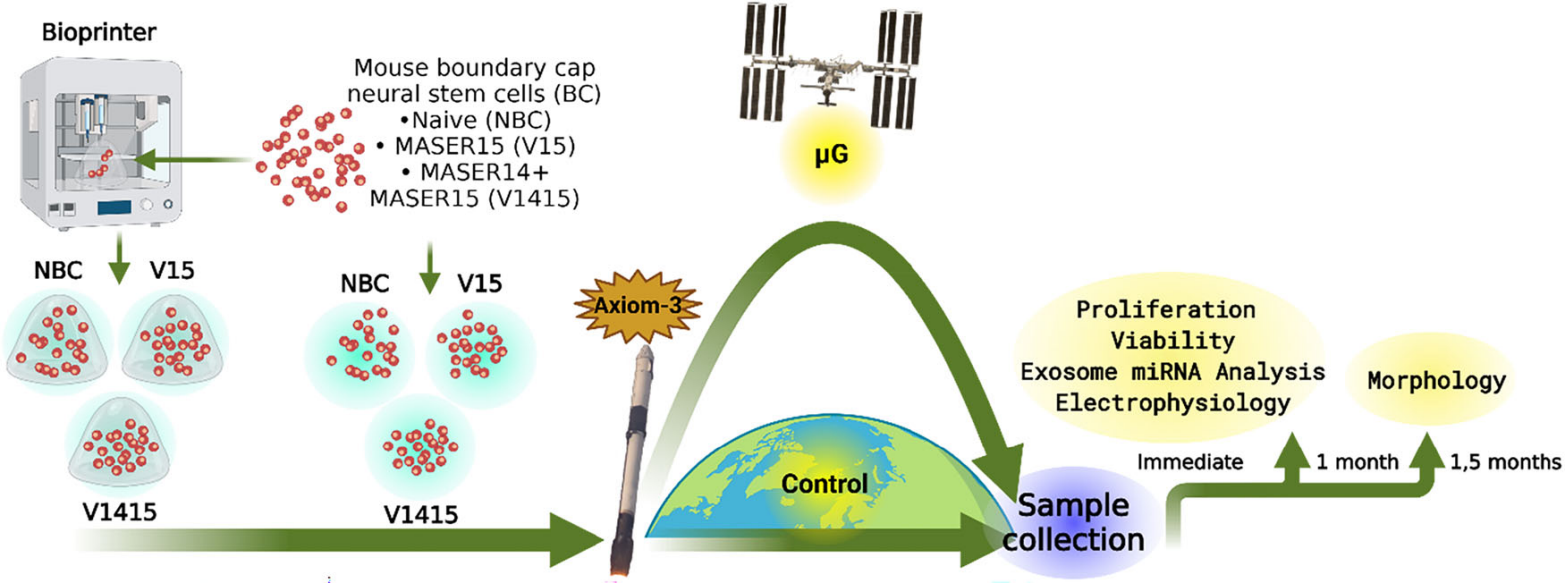
Scheme of the study.

In this study, we investigated the resilience, differentiation potential, and physiological integrity of boundary cap neural crest stem cells (BCs) exposed to the extreme environmental stresses associated with prolonged pre-launch delays and microgravity during an International Space Station (ISS) mission (Fig. 1, Table 1). Despite substantial non-physiological conditions—including more than three weeks outside controlled incubator environments—BCs demonstrated a remarkable capacity for survival and recovery, particularly when supported by 3D-printed bioscaffolds.

**Table 1:** Identification code, group definition and summary of the role of boundary cap neural crest stem cells (BCs) used in the study

Sample ID	Definition	Flight history and role in the study
NBC	Naive pre-flight BCs	BCs never exposed to spaceflight or microgravity. These cells served as the starting population for the current ISS experiment and were not directly analyzed as pre-flight samples in this study.
V15	Once flown pre-flight BCs	BCs previously exposed to short-term microgravity during the MASER 15 sounding rocket mission^22^. These cells served as the starting population for the current ISS experiment and were not directly analyzed as pre-flight samples in this study.
V1415	Twice flown pre-flight BCs	BCs previously exposed to short-term microgravity during both MASER 14^21^ and MASER 15^22^ sounding rocket missions. These cells served as the starting population for the current ISS experiment and were not directly analyzed as pre-flight samples in this study.
FVNBC	Flight naive BCs	Naïve BCs exposed to ISS mission conditions, including long-term microgravity and associated mission-related environmental stress. Surviving cells were recovered and analyzed after landing.
FCNBC	Ground control, naive BCs	Naive BCs maintained on Earth as controls for the FVNBC group. Analyzed in parallel with the corresponding flight-derived cells. *This control was generated from pre-flight NBC cultures and was not a fully mission-matched flight control.*
FV15	Flight, once flown BCs	BCs previously exposed to MASER 15 microgravity^22^ and subsequently flown during the current ISS mission. Samples were collected and analyzed after landing; no viable cells survived for post-flight culture analyses.
FCV15	Ground control, once flown BCs	Ground control cultures corresponding to the FV15 group, maintained on Earth during the ISS mission, and analyzed in parallel after sample recovery; no viable cells survived for culture analysis.
FV1415	Flight, twice flown BCs	BCs previously exposed to MASER 14^21^ and MASER 15^22^ microgravity and subsequently flown during the current ISS mission. Surviving cells were recovered and analyzed after landing.
FCV1415	Ground control, twice flown BCs	Ground control cultures corresponding to the FV1415 group, maintained on Earth during the ISS mission and analyzed in parallel following recovery of the flight samples.

**Table 2:** Summary of exosomal miRNA analysis from the cell culture supernatants

miRNA	Comparison between groups, fold change				Functional axis	Interpretation
	FV1415 - FCV1415	FVNBC - FCNBC	FV1415 - FV15	FVNBC - FV15		
miR-210	4.5	1.3	1	3.1	Hypoxia-responsive miRNA, promotes glycolytic metabolism and adaptation to low oxygen^36^, neuronal plasticity^37^	Increased miR-210 in space lines may indicate switch from oxidative phosphorylation to glycolysis, supporting survival of slowly proliferating cells.
miR-21	4.2	2.5	1.1	4.5	Anti-apoptotic, pro-survival, regulates stress responses^38,39^	Elevated miR-21 may protect space-exposed cells from apoptosis during long-term stress, explaining why FV1415 and FVNBC survive, while the ground controls and FV15 are more prone to cell death.
miR-34a	3.3	2.1	0.9	4.3	Involved in DNA damage response^40^, cell cycle arrest^41^, senescence^42^	Increased miR-34a can drive a shift from fast proliferation to a slow-proliferating, stress-resistant phenotype, fitting with the selection of slowly proliferating survivors in space samples.
miR-221	2.3	1.9	1.1	4.9	Regulates cell cycle, quiescence and adaptation to growth factor deprivation^43^	Stronger miR-221 upregulation in FV1415 (flown twice) may help these cells enter a controlled, slow-cycling state and better adapt to repeated spaceflight and nutrient/oxygen limitations.
miR-17	0.4	0.3	0.6	0.3	Pro-proliferative miRNA cluster, stimulates cell cycle progression and fast growth^44^	Decreased miR-17 may contribute to reduced proliferation rate and selection of slow-proliferating cells that can better tolerate chronic stress in space conditions.
let-7a	0.3	1	0.8	0.9	Controls differentiation and stemness; lower levels often associated with a more plastic, stress-resistant phenotype^45–47^	Lower levels could signify adaptation to stress in flight groups.

Since BCs traveling to the ISS would need to be out of incubator at ambient temperature for a considerable length of time, pre-flight tests were performed to determine the time limit for cell survival and neurosphere production under these conditions. These tests showed an upper limit of three weeks, a time period compatible with the original flight schedule. However, due to delays, the overall time out of incubator exceeded this limit by four days.

Directly after cells were delivered to the laboratory, the samples of medium and cells were collected for genetic and exosome analysis. Microscopic analysis, however, showed that most of retrieved cuvettes only contained cell debris, indicating severe consequences of combined mission-associated stress exposure rather than of isolated microgravity effects. Surviving cells were collected, medium was changed and culture of cells continued after their return to the Uppsala laboratory. When cells reached the sufficient amount for reproducible cultures (around 1 month after landing) morphological and physiological analyses were performed.

Only naive BCs, which had never been exposed to space conditions (FVNBC), and BCs previously flown twice on MASER 14 and MASER 15 (FV1415), together with their corresponding ground controls, survived the space experiment. No viable cells were recovered from the V15 populations under either flight or ground-control conditions (FV15 and FCV15).

For the FVNBC population, no mission-matched ground-control culture survived. Therefore, an additional FCNBC comparison group was generated from pre-flight NBC cells in the Uppsala laboratory and maintained under corresponding ambient-temperature and temporal conditions. This culture does not represent a fully mission-matched ground control.

Recovered FV1415 cells formed neurospheres after return to the laboratory but proliferated markedly slower than observed following previous short-duration sounding rocket missions MASER 14 and MASER 15, or even slower than NBCs not exposed to space experiments. Therefore all post-flight analyses were performed after approximately 1 month of recovery culture, during which surviving cells were expanded to obtain sufficient material for experimentation. Consequently, the analyzed populations represent cells that successfully survived the mission-associated conditions and subsequent recovery period.

The survival of V1415 cells, which had previously experienced two spaceflight missions, raises the possibility that prior exposure to spaceflight-related conditions may be associated with increased resilience during prolonged mission-associated stress. Repeated gravitational unloading may, for example, prime cytoskeletal and stress-response pathways, imparting a “mechanical memory” that supports survival in later microgravity or metabolic stress. This is consistent with the concept of cellular mechanical memory, where past exposure to mechanical stimuli shapes future cell responses through cytoskeletal reorganization and transcriptional regulation^23,24^.

However, the present study was not designed to determine the underlying mechanisms responsible for this observation. Factors such as previous flight history, cumulative handling, storage conditions, passaging history, and other aspects of cell culture history may also have contributed to the observed differences.

Accordingly, while our findings are consistent with a potential preconditioning effect or persistent adaptation resulting from previous spaceflight exposure, we interpret them as an hypothesis rather than direct evidence of cellular mechanical memory or adaptive reprogramming. Additional studies specifically designed to address these mechanisms will be required to establish causality.

### Morphological changes in differentiated BC cultures after exposure to *prolonged mission-associated stress*

**Fig. 2:**
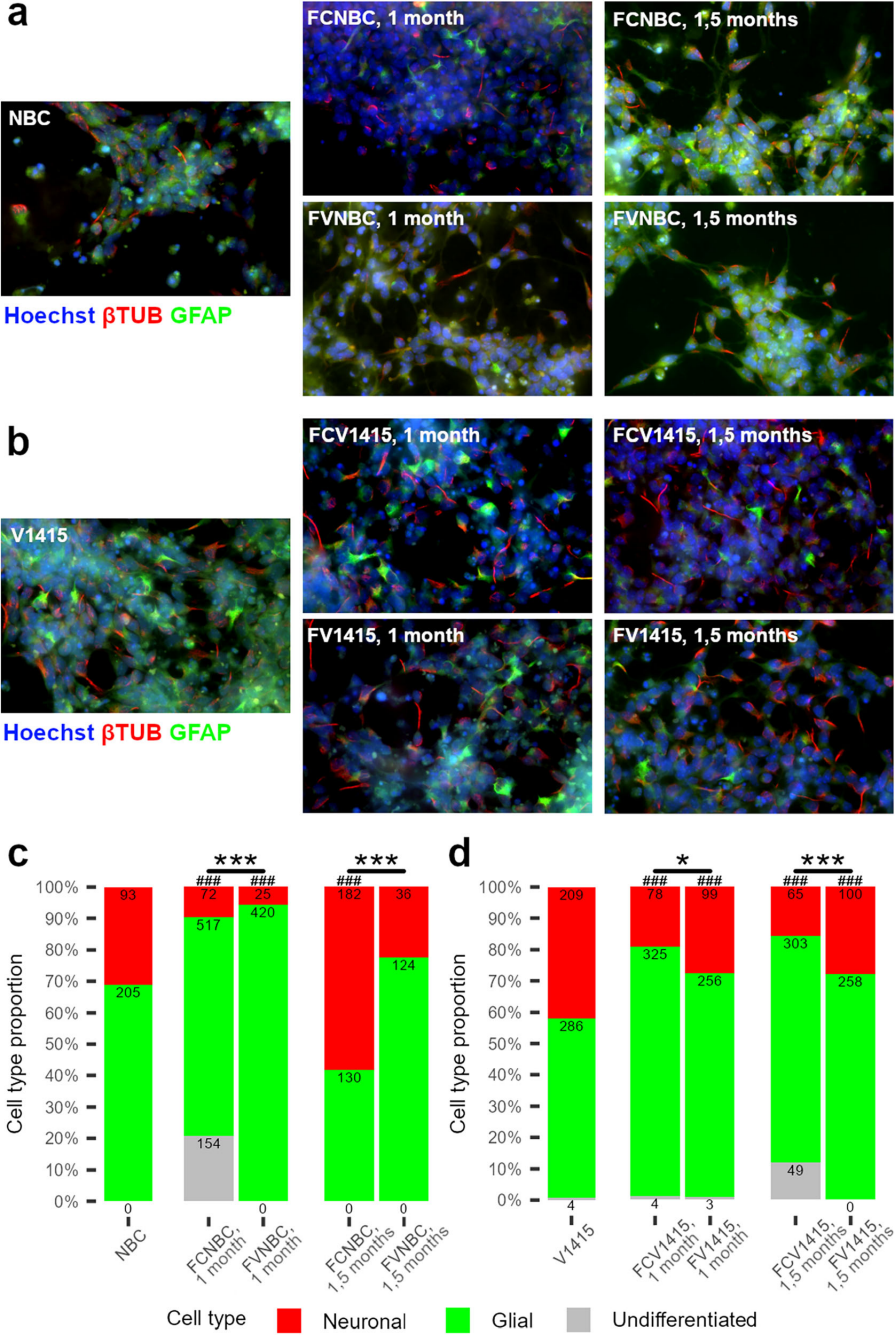
Proportion bar plot of BC differentiation towards neuronal or glial lineages under ground control and flight conditions, calculated from 3 biological replicates. **a** Representative images of experimental groups with NBC cells. **b** Representative images of experimental groups with V1415 cells. **c** Proportions of glial (GFAP^+^βTUB^-^), neuronal (GFAP- βTUB^+^) and undifferentiated cells from NBC cultures (experimental control (NBC), ground control group at 1 month of differentiation (FCNBC, 1 month), spaceflight group at 1 month of differentiation (FVNBC, 1 month), ground control group at 1.5 months of differentiation (FCNBC, 1.5 months), spaceflight group at day 46 of differentiation (FVNBC, 1.5 months)). **d** Proportions for V1415 cultures (experimental control (V1415), ground control group at 1 month of differentiation (FCV1415, 1 month), spaceflight group at 1 month of differentiation (FV1415, 1 month), ground control group at 1.5 months of differentiation (FCV1415, 1.5 months), spaceflight group at 1.5 months of differentiation (FV1415, 1.5 months)). Counts for each cell type noted on or below the bars. Stars denote statistically significant differences between the groups determined by Chi-square test: *p < 0.05, ***p < 0.001. Hashes denote statistically significant differences between the specific group and experimental control: ### *p* < 0.001.

Differentiation assays were performed in all groups of cells 1 month and 1.5 months after culture under normal conditions. We first determined whether the capacity for neuronal and glial differentiation was preserved following prolonged mission-associated stress and subsequent recovery by comparing the proportions of neuronal and glial cells in flight and ground-control groups with their corresponding pre-flight populations. The cultures were labeled with antibodies to βTUB and GFAP, and with Hoechst nuclear stain (Fig. 2a, b).

Cultured cells predominantly differentiated into glial cells. In the NBC group, both pre-flight and post-flight cultures predominantly differentiated into glial cells (Fig. 2c). At the early post-flight time point (1 month), we observed a decreased proportion of neuronal cells in the FVNBC group. However, after 1.5 months, the neuronal proportion was increased in the control group, demonstrating the recovery of FCNBC cultures toward the differentiation profile observed in pre-flight NBC cells. The proportion of neuronal population in cells, subjected to the flight conditions was also increased with time (FVNBC, 1.5 months group)—there were no statistically significant differences when compared to experimental control, demonstrating recovery potential. In the FV1415 group, cells which had already been exposed twice to microgravity, we observed an increased neuronal population in flight samples compared to control samples, both after 1 month and 1.5 months (Fig. 2d), suggesting faster recovery after space-exposed experiment, in the group subjected to the flight condition. This observation suggests that prior cellular history may influence differentiation outcomes following prolonged mission-associated stress, although the mechanisms responsible for this effect remain unknown.

**Fig. 3:**
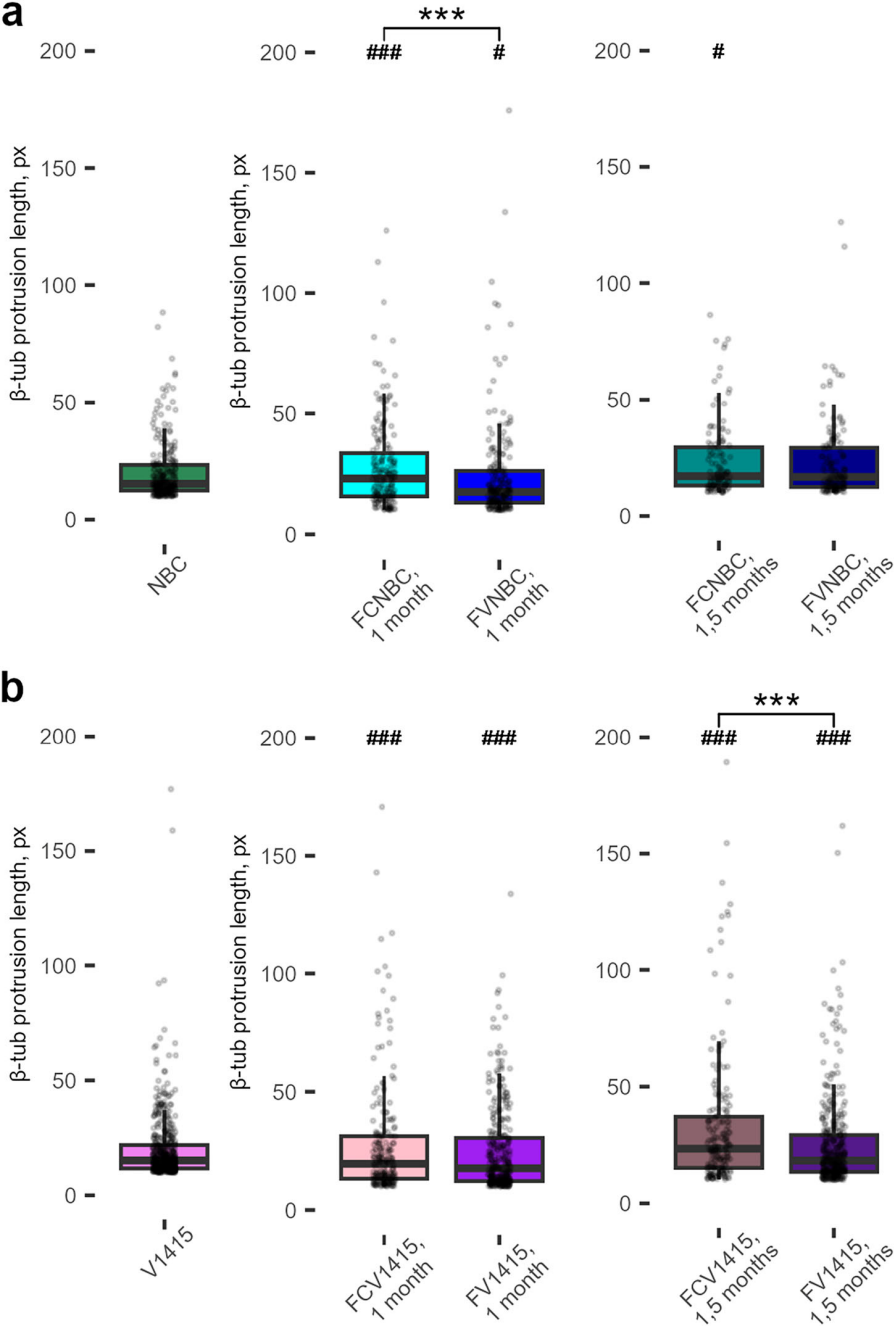
Analysis of BC βTUB protrusion lengths 1 month and 1.5 months after landing, gathered from 3 biological replicates in each group. **a** Analysis of NBC experimental groups. **b** Analysis of V1415 experimental groups. Boxplots and data points displayed, each point representing one protrusion. Stars denote statistically significant differences between control and flight groups, determined by Wilcoxon rank-sum test: ***p < 0.001. Hashes denote statistically significant differences between a specific group and experimental control: #*p* < 0.05, ###*p* < 0.001.

The morphological assessment confirmed that differentiated cells extensively differentiated to neuron and glial population during 2–3 days after 1 month and 1.5 months of culture (Fig. 3). The differentiated progeny displayed stable lineage potential. We next analyzed neurite protrusion lengths of differentiated BCs as an indicator of the degree of neuronal differentiation. Morphological assessment of βTUB-positive cells in all culture groups was performed after 1 and 1.5 months (Fig. 3). The differentiated progeny displayed stable lineage potential. Interestingly, the effects associated with spaceflight exposure differed between the FVNBC and FV1415 populations. FVNBCs developed protrusion lengths similar to those of experimental controls at the 1.5-month time point, with no significant differences observed between groups. In contrast, FV1415 cultures displayed a higher neuronal differentiation than the corresponding FCV1415 controls. This divergence suggests that the cells’ “history” or prior mechanical experiences may also contribute to cell differentiation fate. Moreover, the neuronal bias observed in FV1415 samples may be linked to the differential stress resilience of neural cell populations. Glial cells are known to be more resistant to oxidative and mechanical stress due to their homeostatic and neuroprotective functions^25^, making them more likely to survive adverse conditions. The preferential survival of this population may, in turn, influence the cellular microenvironment and the composition of the remaining progenitor pool. Although neuronal precursors are generally considered more vulnerable to cellular stress because of their reliance on dynamic cytoskeletal remodeling and mitochondrial activity^26,27^, stress exposure can also alter developmental trajectories and promote differentiation of surviving progenitors. Therefore, the increased neuronal differentiation observed in FV1415 cultures may reflect a stress-induced adaptation arising from the selective survival history of the culture rather than a stable shift in lineage commitment.

The observed differences in lineage composition may reflect selective survival or expansion of particular cell subpopulations during the prolonged recovery period, rather than permanent alterations in cell fate commitment. Nevertheless, the maintenance of robust neuronal and glial differentiation across surviving groups demonstrates that BCs retain substantial developmental plasticity despite exposure to conditions exceeding their experimentally determined survival window.

### Physiological assessment

**Fig. 4:**
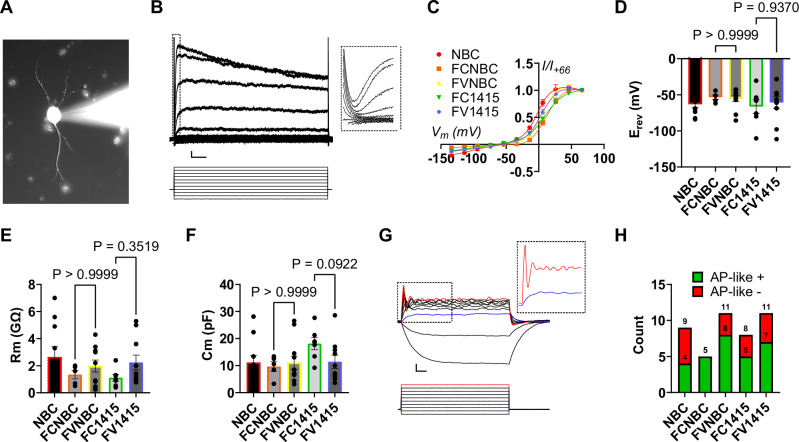
Electrical properties of differentiated NBCs are unaffected by space conditions. **A** Representative image of a patch-clamped differentiated NBC, identified by Alexa Fluor 594 within the internal solution. **B** Representative trace of 20 mV voltage steps and the responding current. Clear rectification is observed. Scale bars represent 20 ms and 100 pA for x and y, respectively. Inset shows a magnification of the slow rise of the current, denoted by dotted lines on the main figure, caused by initial activation of voltage-gated sodium channels, or a delayed activation of voltage-gated potassium channels. **C** Current–voltage (IV) plot of control cells and cells which experienced microgravity. **D** The reversal potential (E_rev_) for control cells and cells which experienced microgravity. No significant differences were observed when comparing the control group to their space-exposed counterparts. **E** The membrane resistance (Rm) of control cells and cells which experienced space conditions. No significant differences were observed when comparing the control group to their space-associated counterparts.**F** Membrane capacitance (Cm) of control cells and cells which experienced microgravity, a measure of membrane surface area. No significant differences were observed when comparing the control group to their space-exposed counterparts. **G** Representative trace of 20 pA current steps, and the corresponding voltage change. Inset shows magnification of the voltage response to depolarizing current steps which did not reach threshold for activation of voltage-gated sodium channels (blue) and a current step that did result in the action potential (AP)-like voltage changes (red). Scale bars represent 20 ms and 10 mV for x and y, respectively. **H** The number of cells within each group which displayed these AP-like voltage changes in response to depolarizing current steps. All graphs are plotted as mean ± SEM, and *P*-values derived from statistical tests are denoted on the graphs.

We next asked if cells subjected to prolonged ambient handling, sealed culture conditions, launch delay, transport, nutrient and gas limitations, and delayed post-flight recovery would display normal physiological properties. To investigate whether these conditions affected the BC’s ability to differentiate into functional neural cells, patch clamp electrophysiology was performed on differentiated cells from NBC, V1415, FCNBC, FVNBC, FC1415 and FV1415 populations. Despite a short amount of time in the differentiation media (2–3 days), most cells were clearly distinct from rounded undifferentiated BCs, many with long processes likely corresponding to immature neuronal or glial populations (Fig. 4a). We initially investigated passive membrane properties by applying voltage steps ranging from −134 mV to +66 mV and measuring the corresponding current changes. A representative current and associated voltage steps are shown in Fig. 4b, with the combined current–voltage (IV) plots shown in Fig. 3c. A clear outwardly rectifying current was observed for every group, indicative of the presence of membrane channels. The reversal potential (E_rev_) was not significantly different between control and space conditions (NBC: −63 ± 5 mV; FCNBC: −53 ± 3 mV; FVNBC: −55 ± 4 mV; FC1415: −66 ± 10 mV; FV1415: −61 ± 8 mV; Fig. 4d). The most likely contributors to these currents are voltage-gated potassium channels, but we cannot discount the presence of chloride and sodium currents. Properties of the cells also did not differ significantly between groups; the membrane resistance (Rm) was similar between groups, suggesting comparable numbers of open channels in the membrane at hyperpolarised potentials (NBC: 2.6 ± 0.8 GΩ; FCNBC: 1.3 ± 0.3 GΩ; FVNBC: 2.0 ± 0.4 GΩ; FC1415: 1.1 ± 0.2 GΩ; FV1415: 2.2 ± 0.5 GΩ; Fig. 4e), and capacitance (Cm) was also similar, suggesting that differentiated cells had similar membrane surface areas (NBC: 11 ± 3 pF; FCNBC: 10 ± 2 pF; FVNBC: 11 ± 2 pF; FC1415: 18 ± 2 pF; FV1415: 11 ± 2 pF; Fig. 4f).

We also examined the excitability of these differentiated BCs using current-clamp. Current steps were applied from -60 pA to +300 pA and membrane voltage recorded (representative image displayed in Fig. 4g). At negative current injection, large hyperpolarising voltage changes were observed, consistent with a large membrane resistance and few open channels. However, at larger positive current injections, action potential-like events were observed, with rapid rise times and fast inactivation, consistent with the actions of voltage-gated sodium channels. Unlike true neuronal action potentials, there was no evidence of a regenerative current, and only a single action potential-like event was observed for each cell, although at higher current injections there were ‘wobbles’ in the voltage traces, consistent with partial recovery from inactivation. These observations are consistent with the voltage clamp data. The magnified portion in Fig. 4b shows the initial rising phase of the current after the fast capacitive transient. The dip before the current rise could be caused either by a delayed action of voltage-gated potassium channels, or the inward current caused by activation of a voltage-gated sodium current, which rapidly desensitizes and is replaced by the outward current of the potassium channels. Cells from all groups showed evidence for this voltage-gated sodium channel activity (Fig. 4h). Taken together, these data indicate that the exposure to space-associated stress conditions did not have significant effects on the electrical properties of these BCs, and that these cells retain similar membrane channels and characteristics.

Importantly, our electrophysiological analyses show that key functional properties (such as voltage-gated sodium and potassium currents, membrane resistance, capacitance, and action-potential-like events) are preserved in flight-derived differentiated cells. This indicates that, despite prolonged environmental stress, BC-derived neurons and glia retain fundamental physiological competence. These findings bolster the idea that BCs are not just surviving, but functionally resilient.

### Relative β-actin genomic DNA abundance

**Fig. 5:**
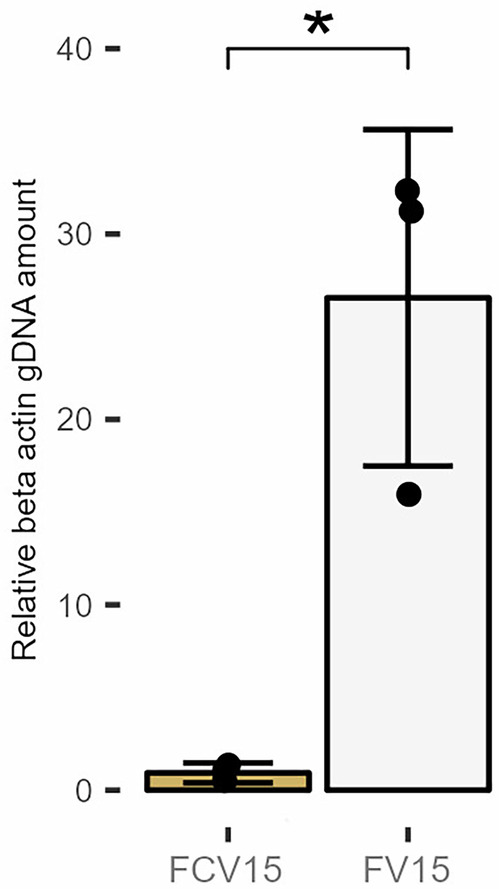
Relative β-actin genomic DNA abundance in FV15 flight and FCV15 ground-control samples. Genomic DNA was analyzed by quantitative PCR (qPCR) targeting the β-actin gene. Relative β-actin genomic DNA abundance was used as an indirect measure of the amount of cell-derived DNA present in the samples. Flight-derived cultures (FV15) exhibited significantly higher β-actin genomic DNA levels than corresponding ground-control cultures (FCV15). Data are presented as means (columns) and SD (crossbars) with individual sample values shown (data points; *N* = 3). Statistical significance (noted with *p < 0.05) was determined by t test.

To estimate the relative amount of cellular material present in the experimental space and ground control samples, quantitative PCR analysis of β-actin genomic DNA was performed. The analysis revealed significantly lower Ct values in flight-derived samples compared with corresponding ground controls, indicating higher levels of amplifiable genomic DNA in the flight cultures. The observed Ct difference of approximately 5 cycles (means and standard deviations of flight-derived and ground control samples equal to 28.796 ± 0.567 and 33.77 ± 1.02, respectively) indicate a significant increase in β-actin genomic DNA abundance in the flight samples (Fig. 5).

Because approximately equal number of cells were initially seeded in all cultures, the increased genomic DNA content detected in the flight samples suggests that the cellular populations followed different trajectories during the mission. However, as the assay provides an indirect measure of DNA abundance, it does not directly distinguish between differences in proliferation, survival, or other factors contributing to the final amount of cellular DNA present in the samples.

One possible interpretation is that BCs in the flight cultures underwent an initial phase of cellular expansion, resulting in increased total cellular biomass and consequently higher genomic DNA content. Such a scenario would be consistent with observations from our previous short-duration sounding rocket missions, where BCs displayed enhanced proliferative activity following exposure to spaceflight conditions. However, in contrast to those earlier studies, the cells recovered from the present mission exhibited markedly reduced proliferation rates after return to the laboratory, and overall survival was low in both flight and ground-control groups. Quiescent stem cell populations have been documented in many contexts, including adult and cancer stem cells, where reduced cycling confers resistance to DNA damage and stress^28,29^.

Taken together, these observations raise the possibility that an early increase in cell number may have been followed by extensive cell loss during the prolonged mission-associated stress period, leaving a selected population of surviving cells characterized by slower proliferation. Under such conditions, elevated β-actin genomic DNA levels would reflect the cumulative cellular biomass generated during the experiment.

Nevertheless, the β-actin qPCR assay provides only an indirect estimate of relative genomic DNA abundance and does not directly measure proliferation kinetics, cell viability, or the timing of cellular expansion and loss. Therefore, the proposed sequence of initial proliferation followed by extensive cellular attrition remains hypothetical and regarded as a biologically plausible interpretation rather than a direct conclusion, which can be made from the present study. Future experiments incorporating longitudinal cell counts and metabolic analyses will be required to test this possibility directly.

### Exosome analysis

Exosomal miRNA analysis revealed a pronounced remodeling of all six selected miRNAs in the space-exposed FV1415 cells compared with their ground control FCV1415 (Table 2). FV1415 exosomes showed a strong upregulation of miR-210, miR-21, miR-34a, and miR-221, accompanied by a marked downregulation of miR-17 and let-7a. This pattern corresponds well with the biological behavior of space cells, which we hypothesize proliferated rapidly during the early phase of spaceflight, while only slowly proliferating cells survived in the later phase. The coexistence in exosomes of miRNAs associated with both early rapid growth and later slow-cycling survival suggests that the exosomal cargo may reflect the sequential selection of distinct cellular subpopulations during space exposure. The comparison between FNBC and its ground control FCNBC displayed a similar, though less pronounced, miRNA modulation. FVNBC exosomes showed a moderate upregulation of miR-210, miR-21, miR-34a, and miR-221 together with a moderate downregulation of miR-17 and let-7a. In contrast, FCNBC—which did not survive—did not exhibit any consistent or directional changes in the six miRNAs. The absence of an adaptive exosomal miRNA pattern in the ground control parallels its failure to remain viable, while FVNBC retained a partial but detectable adaptive signature that corresponds with its survival. Cell line (FV15), which did not survive either in space or on the ground, showed no significant modulation of any of the six miRNAs when compared with its ground control FCV15. Levels of miR-210, miR-21, miR-34a, miR-221, miR-17, and let-7a remained essentially unchanged. This lack of exosomal miRNA remodeling distinguishes FV15 from the two surviving space-exposed cell lines (FV1415 and FVNBC), suggesting that FV15 did not activate any of the molecular programs detectable in exosomes that were associated with survival in the other two lines. Direct comparison of the two space-exposed lines with opposite outcomes—FV1415 (survived) and FV15 (died)—revealed the largest differences in exosomal miRNA expression. FV1415 displayed strong upregulation of miR-210, miR-21, miR-34a, and miR-221 and downregulation of miR-17 and let-7a, whereas FV15 showed no modulation of any of these miRNAs. This contrast defines a clear molecular separation between the highly adapted FV1415 cells and the non-surviving FV15 population.

The most distinctive feature of the surviving space-exposed cells—FV1415 and, to a lesser extent, FVNBC—is the coordinated regulation of miRNAs known to influence oxygen sensing, mitochondrial respiration, ROS homeostasis, and the transition toward glycolytic metabolism. In contrast, FV15, which failed to survive in space and on the ground, did not exhibit any modulation of these pathways, highlighting the central role of mitochondrial and metabolic plasticity in determining cell fate under microgravity. Among the regulated miRNAs, miR-210 strongly upregulated in FV1415, and moderately induced in FVNBC. This suggests that these cell populations rapidly adjusted to a microgravity-induced pseudo-hypoxic state by downregulating mitochondrial respiration. The complete absence of miR-210 induction in FV15 suggests that this line failed to initiate the required mitochondrial remodeling that contributed to its loss of viability. Similarly, the upregulation of miR-34a in FV1415 and FNBC supports the suggestion that mitochondrial quality control mechanisms were activated selectively in the surviving lines. miR-34a targets key regulators of mitochondrial dynamics such as SIRT1 and promotes cell-cycle arrest, mitophagy activation, and stabilization of damaged mitochondria. In contrast, FV15 showed no activation of this pathway, leaving damaged mitochondria unrepaired and energetically compromised. The strong upregulation of miR-21 in FV1415 and FVNBC further supports a survival program centered on controlling mitochondrial apoptosis. miR-21 represses PTEN and enhances AKT signaling, which attenuates mitochondrial permeabilization and reduces caspase-dependent cell death. Again, FV15 lacked this adaptive response entirely. miR-221, moderately upregulated in both surviving lines and may support the formation of slow-proliferating phenotypes capable of sustaining ATP production even under low-oxygen or perturbed gravitational environments. Conversely, the downregulation of miR-17 in FV1415 and FVNBC is consistent with the suppression of rapid proliferation and a shift away from high mitochondrial ATP demand. miR-17 normally enhances proliferation and mitochondrial biogenesis. Similarly, the reduction of let-7a suggests that FV1415 and FNBC adopted a more plastic metabolic identity. FV1415, with the strongest miRNA remodeling, appears to have the strongest metabolic reprogramming strategy. These changes, however, reflect exploratory molecular signatures associated with mission exposure rather than direct evidence of hypoxia or mitochondrial dysfunction. Since no direct metabolic measurements (e.g., oxygen consumption, ATP levels, reactive oxygen species production, or glycolytic activity) were performed, any mechanistic interpretation remains speculative.

### 3D-printed BC cultures: enhanced survival in space-related mission conditions

**Fig. 6:**
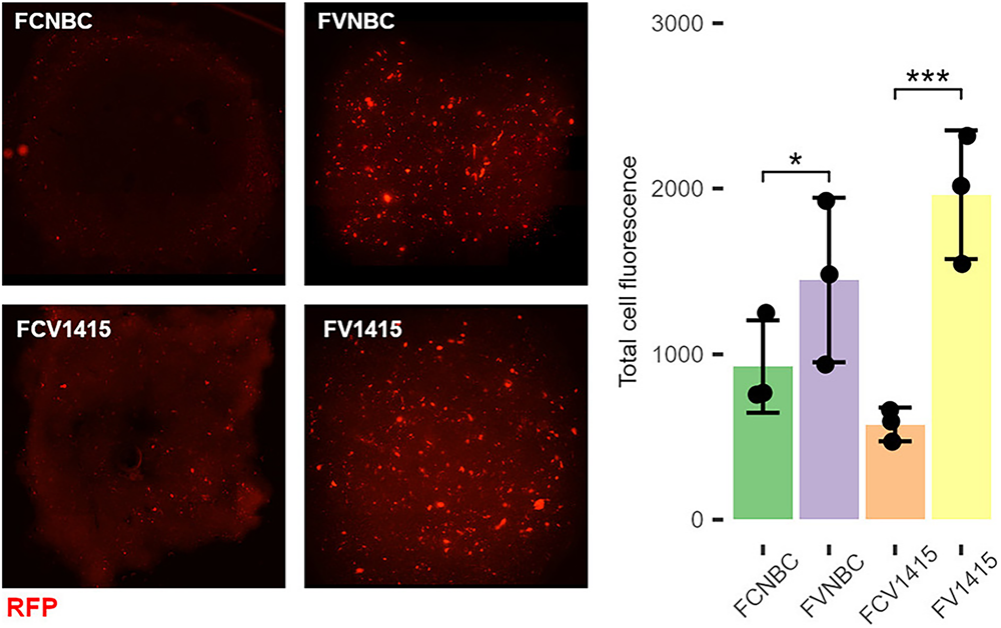
Analysis of total RFP fluorescence of BCs in the bioprinted samples. Representative images displayed on the left. Means (columns), SD (crossbars) and data points displayed. Asterisks denote statistically significant differences between the groups: * *p* < 0.05, *** *p* < 0.001.

BCs embedded in 3D-printed gelatin scaffolds survived better than free-floating neurospheres and were detected in all analyzed scaffold samples from both flight and ground-control groups (Fig. 6). The scaffold-supported cultures maintained cellular integrity throughout the experiment, whereas survival of free-floating neurospheres was substantially reduced.

**Fig. 7:**
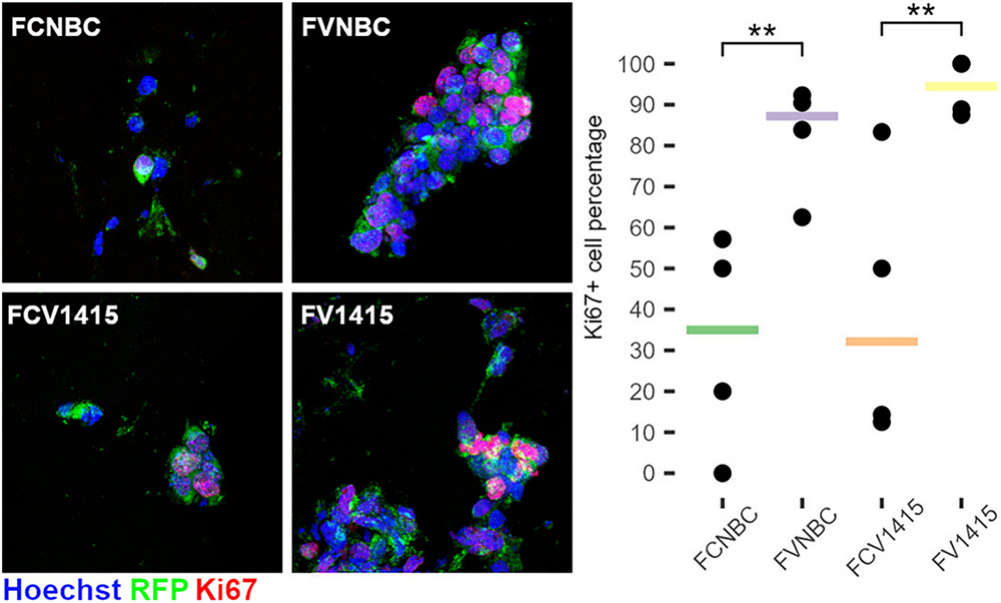
Analysis of Ki67^+^ cell percentage in ground control and spaceflight samples of bioprinted cells. Representative images displayed on the left. Medians (bars) and data points (dots) are displayed. Asterisks denote statistically significant differences between the groups (comprised of 4 images of each cultures), determined by Conover-Iman test: **p < 0.01.

Quantitative analysis demonstrated significantly higher cell numbers in the flight-exposed scaffold samples compared with the corresponding ground controls. Immunohistochemical staining for Ki67 revealed the presence of actively proliferating BCs within the scaffolds of both the FVNBC and FV1415 groups after return from the mission (Fig. 7). The proportion of Ki67-positive cells was particularly prominent in the FV1415 group, indicating preserved proliferative activity despite prolonged exposure to mission-associated stress conditions.

The improved survival and sustained proliferative activity observed in scaffold-supported cultures suggest that the 3D-printed gelatin scaffold may provide a protective microenvironment during prolonged mission-associated stress. The fact that viable and proliferating cells were detected in all scaffold groups, whereas survival of free-floating neurospheres was markedly reduced, supports the concept that three-dimensional culture systems can enhance cellular resilience under challenging environmental conditions. These findings are particularly relevant for future space-based tissue engineering and bioprinting applications, where engineered 3D microenvironments may contribute to prolonged cell survival and maintenance of cellular function during long-duration missions.

## Discussion

In this study, we investigated the resilience, differentiation potential, and physiological integrity of boundary cap neural crest stem cells (BCs) exposed to the extreme environmental stresses associated with an extended mission duration due to launch and return delays and microgravity during an ISS mission. Despite substantial non-physiological—more than three weeks outside controlled incubator environments—BCs demonstrated a remarkable capacity for survival and recovery, particularly when supported by 3D-printed bioscaffolds.

Only naive BCs (FVNBC) and twice-flown BCs (FV1415) survived mission-associated stress, along with their corresponding ground controls. Surviving cells exhibited slower proliferation than in earlier sounding-rocket experiments. (The author EK writes: this comment is repeated many times in the manuscript. I therefore suggest to remove this part of the sentence). Differentiated progeny maintained stable lineage potential. Differences in neuronal and glial proportions were observed depending on flight history; however, we propose, that these differences reflect combined environmental exposure and culture history effects rather than isolated gravitational effects. Analysis of space and ground control samples revealed increased actin DNA signal, suggesting changes in biological material content during space exposure. However, this finding does not directly demonstrate proliferation dynamics and may reflect other biological properties. Exosomal miRNA profiling revealed distinct molecular signatures in surviving space-exposed cells. These signatures are consistent with stress-associated cellular adaptation but remain exploratory and non-mechanistic without direct metabolic or hypoxic measurements.

Electrophysiological analyses demonstrated that BC-derived neural cells retained basic membrane properties, ion channel activity, and excitability-like responses across all groups. Importantly, these findings indicate preservation of fundamental physiological competence of differentiating cells after mission-associated stress exposure.

Importantly, BCs maintained within 3D-printed bioscaffolds showed significantly improved survival in both flight and ground conditions, with evidence of proliferation. This highlights the importance of engineered microenvironments for maintaining stem cell viability under mission-associated stress conditions.

Together, these results highlight the robustness of BCs under combined space mission conditions and underscore the value of engineered 3D culture systems for maintaining stem cell viability during long-duration spaceflight. Future studies should incorporate controlled environmental monitoring (oxygen, temperature, nutrient stability, and metabolic profiling) to disentangle microgravity effects from combined mission-associated stressors.

Taken together, the results demonstrate that selected BC populations can survive prolonged mission-associated environmental stress, including extended periods outside standard incubator conditions combined with spaceflight exposure. The differential survival observed between cell populations suggests that prior cellular history may influence resilience under these conditions, although the specific biological mechanisms remain to be determined.

An unexpected but important outcome of this mission was that the prolonged launch delays extended the total period outside controlled incubator conditions beyond the experimentally established survival window determined during pre-flight testing. Although this substantially reduced overall cell survival, it created a unique opportunity to evaluate BC behavior under conditions that exceeded their anticipated physiological limits. As a result, the present study provides information that would not have been obtained under the originally planned mission timeline.

One notable observation was that surviving BC populations displayed markedly reduced proliferation after recovery compared with the enhanced proliferation previously observed following short-duration sounding-rocket missions. This finding suggests that prolonged mission-associated stress may favor the survival of slower-cycling cellular subpopulations capable of enduring extended environmental challenges. While the mechanisms underlying this response remain unclear, the results demonstrate that BCs can maintain viability, differentiation capacity, and physiological competence despite exposure to conditions exceeding their predicted survival threshold.

These findings have direct implications for the development of future space-based stem-cell culture systems and biomanufacturing platforms. A major rationale for developing 3D-bioprinted tissues and scaffold-supported cultures for space applications is to provide structural and physiological support that more closely resembles native cellular environments. The present results provide experimental evidence supporting this concept, and suggest that engineered 3D microenvironments may represent an important strategy for extending cell survival and maintaining cellular function during long-duration missions.

Our findings suggest that differentiation responses following prolonged mission-associated stress may depend not only on the immediate experimental conditions but also on prior cellular history. The increased neuronal proportion observed in FV1415 cultures compared with FCV1415 controls raises the possibility that repeated exposure to spaceflight-associated conditions may influence subsequent differentiation behavior. Although the present study was not designed to test this directly, the observations are consistent with the hypothesis that previous spaceflight exposure could contribute to a form of cellular adaptation affecting lineage specification. Exosomal miRNA profiling revealed distinct molecular signatures in surviving space-exposed cells. These signatures are consistent with stress-associated cellular adaptation but remain exploratory and non-mechanistic without direct metabolic or hypoxic measurements.

Perhaps the most practically relevant finding was the robust performance of BCs cultured within 3D-printed scaffolds. In contrast to free-floating neurospheres, scaffold-supported cells survived across all experimental groups and retained proliferative activity after return from the mission. The scaffolds likely provide mechanical support, enhance nutrient diffusion, and maintain critical cell–cell/cell–matrix interactions reminiscent of their native niche^30^. Such architectural support may help counteract the disruptive effects of microgravity and space-associated stress conditions on cytoskeletal tension and mechanotransduction. These data align with prior evidence that three-dimensional culture systems improve stem cell viability and function by better recapitulating in vivo mechanical and biochemical cues^31,32^. These observations suggest that three-dimensional engineered microenvironments provide substantial protection under prolonged mission-associated stress conditions. Importantly, this protective effect became evident only because the cells were challenged beyond the survival limits established during pre-flight testing. Had the mission proceeded according to the original schedule, the advantages of scaffold-supported culture might have remained undetected.

Taken together, these observations raise the possibility that the lineage shifts detected after recovery culture may represent adaptive or survival-associated differentiation trajectories rather than permanent alterations in cell fate commitment. Future studies specifically designed to investigate the molecular basis of these responses will be required to distinguish between selective survival, cellular adaptation, and other potential mechanisms.

## Methods

### Culture of boundary cap neural crest stem cells (BCs)

Boundary cap neural crest stem cells (BCs) were isolated from an embryo of transgenic mice harboring red fluorescent protein (RFP) under the universal actin promoter^33^ according to previously published protocols^2,3^. Dorsal root ganglia along with boundary caps were mechanically separated from the isolated spinal cord and mechano-enzymatically dissociated using Collagenase/Dispase (1 mg/ml) and DNase (0.5 mg/ml) for 30 min at room temperature. Cells were plated at 0.5–1 × 10^5^ cells/cm^2^ in an N2 medium (Thermo Fisher Scientific, 17502048) containing B27 (Thermo Fisher Scientific, 17504044) as well as EGF (R&D Systems, 236-EG) and bFGF (R&D Systems, 3718-FB), 20 ng/ml, respectively. After 12 h, non-adherent cells were removed together with half of the medium before adding fresh medium. The medium was changed every other day (50% of the medium replaced with fresh medium) until neurospheres could be observed after approximately three weeks of culture. Non-passaged neurospheres between two and three weeks in culture were frozen and stored in liquid nitrogen. All spaceflight experiments were performed using cultures derived from the same original NBC population. The experimental groups were designated according to their previous flight history and experimental background (Table 1).

After return from the ISS the medium was exchanged at the Kennedy Space Center, and the cells were delivered to the Uppsala laboratory. No surviving cells were detected in the V15 groups. Small fragments of BC neurospheres were detected in the V1415 groups in both space (FV1415) and ground (FCV1415) conditions, as well as in the space NBC groups (FVNBC). The spheres were transferred to fresh proliferation medium and cultured for approximately 1 month with regular passaging and medium exchange until the required confluence was reached.

Cells were harvested for morphological and physiological analyses after approximately 1 and 1.5 months of culture, respectively, and the remaining cells were cryopreserved in liquid nitrogen. Because the FVNBC cultures survived the spaceflight mission whereas the corresponding ground-control cultures maintained at the Kennedy Space Center did not survive, an additional control group was generated from pre-flight NBC cultures and maintained in the Uppsala laboratory under comparable non-incubator conditions at ambient temperature for the same duration as the flight experiment. This group represents a non-mission-matched laboratory control, although it was not subjected to the same mission-associated handling, transport, and environmental conditions as the flight samples.

### Description of the experimental groups

Three categories of BC specimens were sent to space:

- NBC = naive BCs never exposed to space conditions (the source for V15 and V1415 cultures).
- V15 = once-flown BCs (on MASER 15 Sounding Rocket^22^, expended after MASER 15 mission and stored in liquid nitrogen before the current experiment).
- V1415 = twice-flown BCs (on MASER 14^21^ and on MASER 15^22^ Sounding Rockets.

The V14 cultures used for MASER 15 mission and therefore designated as V1415.

Expended after MASER 15 mission and stored in liquid before the current experiment).

The flight and ground-control groups were designated according to their respective experimental histories, as summarized in Table 1.

Ground and flight samples experienced shared mission-associated environmental constraints including prolonged pre-launch handling, sealing, and transport; however, exact environmental matching conditions were not fully identical and are considered as a limitation.

### Exosome isolation

Exosomes from all BC populations included in the study were isolated from conditioned medium, which was collected directly in the Kennedy Space Center laboratory and sent on ice to the Einstein College of Medicine, New York, USA. The Cell Culture Media Exosome Purification Kit (Norgen Biotek Corp., Thorold, ON, Canada) was used for exosome isolation, following the manufacturer’s instructions. Briefly, the required volume of cell culture medium was harvested and centrifuged at 200 × *g* for 15 min to remove cells and debris, after which the clarified supernatant was transferred into a fresh conical tube. For every 10 mL of cell-free medium, 25 µL of ExoC Buffer and subsequently 200 µL of Slurry E were added, and the mixture was vortexed for 10 s and incubated at room temperature for 5 min before being vortexed again and centrifuged for 2 min at 2000 RPM. The supernatant was discarded, and an additional brief centrifugation was performed to collect residual liquid, which was carefully removed without disturbing the pellet. The pellet was then resuspended in 200 µL of ExoR Buffer, vortexed for 10 s, and incubated for 5 min at room temperature. After incubation, the suspension was vortexed again for 10 s and centrifuged for 2 min at 500 RPM. The resulting supernatant was transferred to a Mini Filter Spin Column fitted with a 2 mL collection tube and centrifuged for 1 min at 6000 RPM to obtain the final flow-through containing purified exosomes, which were stored at −20 °C until further use.

### Total exosome RNA extraction and miRNA sequencing

Exosomal RNA was extracted from previously isolated exosomes using the Total Exosome RNA and Protein Isolation Kit (Thermo Fisher Scientific), following the manufacturer’s protocol. A total of 100 ng of purified RNA, comprising both long RNAs and small RNA species including miRNAs, was eluted in 20 μL of Elution Solution. Profiling of exosomal miRNA expression was performed using miScript miRNA PCR Arrays (Qiagen, MBRN-105Z), designed to assess the expression of the 84 most highly abundant miRNAs. For first-strand cDNA synthesis, 200 ng of total RNA per sample were reverse-transcribed using the miScript II Reverse Transcription Kit (Qiagen) in accordance with the manufacturer’s instructions. The resulting cDNA was subjected to a pre-amplification step using the miScript PreAMP PCR Kit, after which it was combined with QuantiTect SYBR Green PCR Master Mix, miScript Universal Primer, and RNase-free water. Aliquots of 25 μL of the prepared PCR mixture were dispensed into each well of the miRNA array plate. Quantitative PCR was conducted using a Rotor-Gene Q 100 real-time PCR system (Qiagen) under the following thermal cycling conditions: an initial enzyme activation step at 95 °C for 15 min, followed by 40 amplification cycles consisting of denaturation at 94 °C for 15 s, primer annealing at 55 °C for 30 s, and extension at 70 °C for 30 s. Relative miRNA expression levels were calculated using the 2⁻^ΔΔCt^ method, normalized to the internal controls provided in the array kit and compared with reference control samples. MiRNAs exhibiting a fold change greater than 2 or less than 0.5, together with a *p*-value below 0.05, were considered significantly differentially expressed.

### Bioinformatic and statistical analysis of miRNAs

miRNAs from QIAseq miRNA-NGS data analysis software were selected based on read number. The final list of miRNAs obtained was used in enrichment analysis using miRNet software (Xia Lab, https://www.mirnet.ca/). Functional enrichment analysis of miRNA was realized using miRTareBase v8.0 database as reference. The software was exploited to perform a Gene Ontology Biological Process Enrichment. A *p*-value < 0.05 was chosen to select data, and Prism 8.03 software graphical view (GraphPad Software Inc., Boston, MA, United States) was used to report enrichment analysis.

### Scaffold preparation

Prior to bioprinting, BC neurospheres were gently collected using a 10 mL pipette and transferred to a 14 mL centrifuge tube. The neurospheres were allowed to sediment by gravity at room temperature for approximately 4–5 min, depending on their size. After sedimentation, the supernatant was carefully removed, and the pellet was resuspended in 4 mL of N2 medium. The neurospheres were then returned to the same well of a low-attachment 6-well plate. The culture medium was replaced every other day until the neurospheres were mixed with the bioink immediately prior to scaffold printing.

The printing process was performed according to a previously reported procedure^34,35^. A 10% gelatin solution was warmed to 37 °C and combined with 10% 30 mg/mL microbial transglutaminase (mTG) and 10 µg/mL laminin (BioLamina, LN521) to obtain a final 4% gelatin–cell mixture. The bioink was loaded into a 3 mL luer-lock syringe, cooled at 4 °C for around 10 min until it became Newtonian fluid state. Single cells were then incorporated using two syringes connected by a luer-lock adapter. The final mixture was attached to the GESIM BioScaffolder 3.3 PRIME printer with a 4 °C temperature-controlled head and printing bed. Three-layer scaffolds were printed directly into 12-well plates, after which culture medium was added and scaffolds were transferred to the incubator for further culture.

The bioink was developed and characterized through rheological analyses and formulated as a mechanically stable, cell-compatible hydrogel designed to provide extracellular matrix-like support during extended culture and exposure to mission-associated stress conditions^35^. Prior to its application in the spaceflight experiments, the bioink was evaluated in long-term in vitro studies and demonstrated its ability to support BC survival, proliferation, and differentiation for at least 1 month in culture^35^.

Following completion of the ISS experiment, both flight and ground-control scaffolds were returned to the Uppsala laboratory and immediately fixed in 4% paraformaldehyde (PFA) at room temperature. The samples were subsequently cryoprotected overnight in 30% sucrose, embedded in Tissue-Tek® O.C.T. compound (Sakura Finetek), and rapidly frozen on dry ice. Cryosections (12-μm thick) were prepared and stored in cold for subsequent immunohistochemical processing.

### Preparation and assembly of cell cultures at the Kennedy Space Center

Prior to the Axiom-3 experiment, three BC specimen categories—NBC, V15, and V1415—were thawed and expanded in the Uppsala laboratory for approximately three weeks to obtain sufficient cell numbers for the spaceflight experiment. The expanded cultures were then cryopreserved in multiple aliquots and transported in a frozen state to the Kennedy Space Center.

Three days before launch, cells were thawed and prepared for both the spaceflight experiment and the corresponding ground controls, which were maintained on the laboratory bench at ambient temperature for the entire duration of the space experiment, without medium exchange, under conditions identical to the flight samples.

Experimental BCs were either suspended as free-floating neurospheres in proliferation medium or seeded within 3D-printed bioscaffolds containing proliferation medium.

Flight and ground-control samples were prepared simultaneously. For each experimental group, cells were derived from the same well of a 6-well culture plate and distributed in parallel between flight and ground-control conditions to minimize variability arising from preparation procedures or initial cell population differences.

Around 50 neurospheres were placed into 100 × 3 mL cuvettes with tightly closed lids, completely wrapped with parafilm and assembled in a custom-designed ICE Cubes experimental 3D-printed box, ensuring tight positioning of the specimens. The box with the cells was delivered 48 h before scheduled launch to the SpaceX Dragon 2 spacecraft (https://www.spacex.com/vehicles/dragon/) at the Kennedy Space Center (https://www.nasa.gov/kennedy/). Due to technical issues, spacecraft launch was postponed for 24 h, leaving cells in a pre-launch state for altogether 72 h.

Given the outcome in terms of survival following the Axiom Mission 3 (see “Results”) an additional group of NBC ground control for comparison with surviving FVNBC were included in the post-flight analysis at the Uppsala laboratory.

Ground controls were maintained in parallel, but were not exposed to the same thermal fluctuations, atmospheric conditions, or transport environment as the flight samples; therefore, comparisons reflect mission-associated environmental and handling stresses rather than strictly matched space conditions.

### Quantification of β-actin genomic DNA

To estimate the relative amount of cellular material present in space and ground control samples, genomic DNA was isolated and analyzed by quantitative PCR (qPCR) targeting the β-actin gene. Amplification reactions were performed in duplicate, and threshold cycle (Ct) values were recorded. Relative differences in β-actin genomic DNA abundance between samples were estimated from Ct value differences using standard qPCR assumptions. Because equal numbers of cells were initially seeded in the flight and ground-control cultures, β-actin genomic DNA abundance was used as an indirect measure of relative cellular content in the experimental samples.

### Differentiation assay

Differentiation assays were performed to evaluate the capacity of boundary cap neural crest stem cells (BCs) to generate neuronal and glial progeny following recovery from the experimental conditions. Neurospheres were plated onto glass coverslips coated with poly-L-lysine (PLL) and laminin. Briefly, coverslips were coated with 0.01% PLL for 1–2 h at room temperature, rinsed with sterile water, and subsequently coated with laminin (10 μg/mL) for 1–2 h at 37 °C. Neurospheres were transferred to the coated coverslips in differentiation medium and allowed to attach and initiate differentiation. The medium was refreshed after two days to support cell survival and differentiation. After three days of differentiation, cultures were fixed in 4% paraformaldehyde (PFA) for subsequent immunocytochemical analyses.

Scaffolds from all experimental groups were maintained in proliferation medium throughout the mission and until their return to the laboratory in Uppsala. Upon arrival, scaffolds were carefully removed from the culture chambers, fixed in 4% PFA, cryoprotected overnight in 30% sucrose, embedded in Tissue-Tek® O.C.T. compound, rapidly frozen on dry ice, and sectioned at 12 μm thickness using a cryostat.

Sections for immunohistochemical and immunocytochemical analyses were incubated overnight at 4 °C, using antibodies against βIII-tubulin (βTUB; mouse, 1:400, ZYMED) as a neuronal marker, antibodies to glial fibrillary acidic protein (GFAP; rabbit, 1:400, Agilent Dako) as a glial marker, and Ki67 as a proliferation marker (Invitrogen, AB_2574235). Secondary antibodies were Alexa Fluor 488 goat anti-rabbit IgG (H + L; 1:200, Life Technologies) and FITCgoat anti-mouse IgF (H + L; 1:200, ImmunoResearch). Cell nuclei were visualized using Hoechst nuclear stain (Invitrogen, 2433875, 1:4000). Stained cultures and scaffold sections were examined and documented using fluorescence microscopy, and the proportions of neuronal, glial, and proliferating cells were quantified for comparative analyses between experimental groups.

### Image analysis

Fluorescently labeled cultures were imaged to quantify cell populations and assess the degree of differentiation. Images were analyzed using ImageJ (version 1.52a). The number of GFAP-positive (green) and βIII-tubulin (βTUB)-positive (red) cells was determined using the Analyze Particles function after masking the nuclei based on the Hoechst (blue) signal. To quantify neuronal morphology, βTUB-positive protrusion lengths were measured by masking the βTUB signal and applying the Ridge Detection plugin in ImageJ, with the minimum fiber length set to 10 px.

### Electrophysiology

Differentiated BCs were identified based on their characteristic morphology. Cells were recorded using an Axopatch 200B or 700B amplifier and digitized using a Digidata 1440A. Data were acquired using Clampex Ver. 10.6. Recordings were sampled at 20 kHz, and low-pass filtered at 2 kHz. All recordings were performed at room temperature.

BCs were recorded using borosilicate glass electrodes with resistances 5–7 MΩ when filled with an internal solution containing (in mM): 130 K-gluconate, 2 NaCl, 0.01 CaCl_2_, 2 MgCl_2_, 10 HEPES, 0.01 BAPTA, 2 Na_2_ATP, 0.5 Na_2_GTP, 10 phosphocreatine (pH 7.2). Osmolarity was 300 ± 20 mOsm/L. Internal solution was supplemented with Alexa Fluor 594 (100 μM) for identification of cell morphology. Cells were perfused with a modified Kreb’s solution containing (in mM): 144 NaCl, 1 NaH_2_PO_4_, 2.5 KCl, 10 HEPES, 2 MgCl_2_, 2 CaCl_2_, 10 glucose (pH 7.3). The junction potential was ~14 mV, and cells were held at −74 mV.

Voltage and current clamp experiments used steps of 20 mV and 20 pA, respectively. Current–voltage (IV) plots were created by measuring the steady-state current at each voltage step. To account for variety in channel expression and membrane surface area between cells, the steady-state current was normalized to the current measured at the highest voltage (66 mV). The reversal potential (E_rev_) was estimated by calculating the x-intercept of a plotted straight line on the IV curve.

Action potential-like events were visually identified during current-clamp experiments, based on the typical voltage-gated sodium channel current (fast rise time followed by rapid desensitization and partial fluctuating recovery for the duration of the current injection). Membrane resistance (Rm) was calculated using Ohm’s law (R = ΔV/ΔI; where R is the resistance, ΔV is the magnitude of the voltage step, and ΔI is the change in steady-state current). Membrane capacitance (Cm) was estimated by measuring charge transfer (Q) during a voltage step according to the equation Cm = Q/ΔV.

### Statistical analyses

A significance level of α = 0.05 was used for all analyses. Differences between the proportions of GFAP⁺βTUB⁻, GFAPβTUB⁺, and GFAP⁻βTUB⁻/GFAP⁻βTUB⁺ cells were assessed using the Chi-square test. Data distribution was evaluated with the Shapiro–Wilk test to determine normality. For normally distributed data, comparisons between two groups were performed using an unpaired t-test, while non-normally distributed data were analyzed using the Wilcoxon test. Significance of differences between the experimental groups of the bioprinted samples were determined via Conover-Iman test. Data from electrophysiological experiments were analyzed using Clampfit ver. 10.6. Data were plotted and statistical analysis performed in GraphPad Prism (ver. 10.4.2). Data were analyzed and compared using Kruskal–Wallis tests. The control and corresponding microgravity groups were compared using Dunn’s multiple comparisons test.

## Data Availability

The datasets generated and analyzed during the current study are not publicly available because they comprise heterogeneous experimental datasets, including imaging, electrophysiological, molecular, and cell culture data generated using different analytical platforms, requiring specialized processing and interpretation. The datasets are available from the corresponding author upon reasonable request.

## References

[1] Maro, G. S. et al. Neural crest boundary cap cells constitute a source of neuronal and glial cells of the PNS. *Nat. Neurosci.* **7**, 930–938 (2004). 15322547 10.1038/nn1299

[2] Hjerling-Leffler, J. et al. The boundary cap: a source of neural crest stem cells that generate multiple sensory neuron subtypes. *Development* **132**, 2623–2632 (2005). 15872002 10.1242/dev.01852

[3] Aldskogius, H. et al. Regulation of boundary cap neural crest stem cell differentiation after transplantation. *Stem Cells* **27**, 1592–1603 (2009). 19544468 10.1002/stem.77 PMC2733376

[4] Aquino, J. B. et al. In vitro and in vivo differentiation of boundary cap neural crest stem cells into mature Schwann cells. *Exp. Neurol.* **198**, 438–449 (2006). 16442526 10.1016/j.expneurol.2005.12.015

[5] Zujovic, V. et al. Boundary cap cells are highly competitive for CNS remyelination: fast migration and efficient differentiation in PNS and CNS myelin-forming cells. *Stem Cells* **28**, 470–479 (2010). 20039366 10.1002/stem.290

[6] Trolle, C. et al. Boundary cap neural crest stem cells homotopically implanted to the injured dorsal root transitional zone give rise to different types of neurons and glia in adult rodents. *BMC Neurosci.* **15**, 60 (2014). 24884373 10.1186/1471-2202-15-60 PMC4055944

[7] Radomska, K. J. & Topilko, P. Boundary cap cells in development and disease. *Curr. Opin. Neurobiol.* **47**, 209–215 (2017). 29174469 10.1016/j.conb.2017.11.003

[8] Olerud, J. et al. Neural crest stem cells increase beta cell proliferation and improve islet function in co-transplanted murine pancreatic islets. *Diabetologia* **52**, 2594–2601 (2009). 19823803 10.1007/s00125-009-1544-z

[9] Grouwels, G. et al. Differentiating neural crest stem cells induce proliferation of cultured rodent islet beta cells. *Diabetologia* **55**, 2016–2025 (2012). 22618811 10.1007/s00125-012-2542-0

[10] Ngamjariyawat, A., Turpaev, K., Vasylovska, S., Kozlova, E. N. & Welsh, N. Co-culture of neural crest stem cells (NCSC) and insulin producing beta-TC6 cells results in cadherin junctions and protection against cytokine-induced beta-cell death. *PLoS ONE* **8**, e61828 (2013). 23613946 10.1371/journal.pone.0061828 PMC3629122

[11] Ngamjariyawat, A., Turpaev, K., Welsh, N. & Kozlova, E. N. Coculture of insulin-producing RIN5AH cells with neural crest stem cells protects partially against cytokine-induced cell death. *Pancreas* **41**, 490–492 (2012). 22415669 10.1097/MPA.0b013e31823fcf2a

[12] Grapensparr, L. et al. Co-transplantation of human pancreatic islets with post-migratory neural crest stem cells increases β-cell proliferation and vascular and neural regrowth. *J. Clin. Endocrinol. Metab.* **100**, E583–E590 (2015). 25668197 10.1210/jc.2014-4070

[13] Lau, J., Vasylovska, S., Kozlova, E. N. & Carlsson, P. O. Surface coating of pancreatic islets with neural crest stem cells improves engraftment and function after intraportal transplantation. *Cell Transpl.* **24**, 2263–2272 (2015). 10.3727/096368915X686184 25581301

[14] Aggarwal, T. et al. Hoeber, J., Ivert, P., Vasylovska, S. & Kozlova, E.N. Boundary cap neural crest stem cells promote survival of mutant SOD1 motor neurons. *Neurotherapeutics* **14**, 773–783 (2017). 28070746 10.1007/s13311-016-0505-8 PMC5509618

[15] Schizas, N. Neural crest stem cells protect spinal cord neurons from excitotoxic damage and inhibit glial activation by secretion of brain-derived neurotrophic factor. *Cell Tissue Res* **372**, 493–505 (2018). 29516218 10.1007/s00441-018-2808-z PMC5949140

[16] Leyton-Jaimes, M. F. et al. Empty mesoporous silica particles significantly delay disease progression and extend survival in a mouse model of ALS. *Sci. Rep.* **10**, 20675 (2020). 33244084 10.1038/s41598-020-77578-x PMC7691331

[17] Shahzad, Z., Rafay, R. H., Bala, N., Dogan, Y. E. & Alli, A. A. The heart in space: effects of microgravity on different cell types and their functions in the cardiovascular system. *Biomedicines* **13**, 2336 (2025). 41153622 10.3390/biomedicines13102336 PMC12562242

[18] Cheng, K. et al. Simulated microgravity reduces quality of ovarian follicles and oocytes by disrupting communications of follicle cells. *NPJ Microgravity* **9**, 7 (2023). 36690655 10.1038/s41526-023-00248-5 PMC9870914

[19] López Garzón, N. A. et al. Microgravity and cellular biology: insights into cellular responses and implications for human health. *Int. J. Mol. Sci.* **26**, 3058 (2025). 40243850 10.3390/ijms26073058 PMC11988870

[20] Mozneb, M. et al. Stem cell research in space: advancing regenerative medicine beyond Earth. *Cell Stem Cell* **32**, 1491–1508 (2025). 41043403 10.1016/j.stem.2025.09.001

[21] Han, Y. et al. Molecular genetic analysis of neural stem cells after space flight and simulated microgravity on Earth. *Biotechnol. Bioeng.* **118**, 3832–3846 (2021). 34125436 10.1002/bit.27858

[22] Han, Y. et al. Effects of microgravity on neural crest stem cells. *Front. Neurosci.* **18**, 1379076 (2024). 38660221 10.3389/fnins.2024.1379076 PMC11041629

[23] Dudaryeva, O. Y., Bernhard, S., Tibbitt, M. W. & Labouesse, C. Implications of cellular mechanical memory in bioengineering. *ACS Biomater. Sci. Eng.* **9**, 5985–5998 (2023). 37797187 10.1021/acsbiomaterials.3c01007 PMC10646820

[24] Matellan, C. & Del Río Hernández, A. E. Engineering the cellular mechanical microenvironment—from bulk mechanics to the nanoscale. *J. Cell Sci.* **132**, jcs229013 (2019). 31040223 10.1242/jcs.229013

[25] Bhatia, T. N. et al. Astrocytes do not forfeit their neuroprotective roles after surviving intense oxidative stress. *Front. Mol. Neurosci.* **12**, 87 (2019). 31024254 10.3389/fnmol.2019.00087 PMC6460290

[26] Attwell, D. & Laughlin, S. B. An energy budget for signaling in the grey matter of the brain. *J. Cereb. Blood Flow. Metab.* **21**, 1133–1145 (2001). 11598490 10.1097/00004647-200110000-00001

[27] Harris, J. J., Jolivet, R. & Attwell, D. Synaptic energy use and supply. *Neuron* **75**, 762–777 (2012). 22958818 10.1016/j.neuron.2012.08.019

[28] Moore, N. & Lyle, S. Quiescent, slow-cycling stem cell populations in cancer: a review of the evidence and discussion of significance. *J. Oncol.* **2011**, 396076 (2011). 20936110 10.1155/2011/396076 PMC2948913

[29] Pisanu, M. E. et al. Lung cancer stem cell lose their stemness default state after exposure to microgravity. *Biomed. Res. Int.* **2014**, 470253 (2014). 25276790 10.1155/2014/470253 PMC4170742

[30] Bektas, C. K., Luo, J., Conley, B., Le, K. N. & Lee, K. B. 3D bioprinting approaches for enhancing stem cell-based neural tissue regeneration. *Acta Biomater.* **193**, 20–48 (2025). 39793745 10.1016/j.actbio.2025.01.006

[31] Marchini, A. & Gelain, F. Synthetic scaffolds for 3D cell cultures and organoids: applications in regenerative medicine. *Crit. Rev. Biotechnol.* **42**, 468–486 (2022). 34187261 10.1080/07388551.2021.1932716

[32] Meng, X., Leslie, P., Zhang, Y. & Dong, J. Stem cells in a three-dimensional scaffold environment. *Springerplus* **3**, 80 (2014). 24570851 10.1186/2193-1801-3-80 PMC3931863

[33] Vintersten, K. et al. Mouse in red: red fluorescent protein expression in mouse ES cells, embryos, and adult animals. *Genesis* **40**, 241–246 (2004). 15593332 10.1002/gene.20095

[34] Han, Y. et al. Towards 3D bioprinted spinal cord organoids. *Int. J. Mol. Sci.* **23**, 5788 (2022). 35628601 10.3390/ijms23105788 PMC9144715

[35] Han, Y. et al. Differentiation of iPSC-derived neural progenitors into motor neurons in 3D-printed bioscaffolds. *Int. J. Bioprint.* **11**, 498–515 (2025).

[36] Preda, M. B. et al. miR-210 locus deletion disrupts cellular homeostasis: an integrated genetic study. *Sci. Rep.* **15**, 22659 (2025). 40595052 10.1038/s41598-025-07572-8 PMC12217331

[37] Watts, M., Williams, G., Lu, J., Nithianantharajah, J. & Claudianos, C. MicroRNA-210 regulates dendritic morphology and behavioural flexibility in mice. *Mol. Neurobiol.* **58**, 1330–1344 (2021). 33165828 10.1007/s12035-020-02197-6

[38] Chawra, H. S. et al. MicroRNA-21’s role in PTEN suppression and PI3K/AKT activation: Implications for cancer biology. *Pathol. Res. Pract.* **254**, 155091 (2024). 38194804 10.1016/j.prp.2024.155091

[39] Xie, J. et al. Roles of microRNA-21 in skin wound healing: a comprehensive review. *Front. Pharmacol.* **13**, 828627 (2022). 35295323 10.3389/fphar.2022.828627 PMC8919367

[40] Kofman, A. V. et al. microRNA-34a promotes DNA damage and mitotic catastrophe. *Cell Cycle* **12**, 3500–3511 (2013). 24091633 10.4161/cc.26459 PMC3906336

[41] Yamakuchi, M., Ferlito, M. & Lowenstein, C. J. miR-34a repression of SIRT1 regulates apoptosis. *Proc. Natl. Acad. Sci. USA* **105**, 13421–13426 (2008). 18755897 10.1073/pnas.0801613105 PMC2533205

[42] Xu, X. et al. miR-34a induces cellular senescence via modulation of telomerase activity in human hepatocellular carcinoma by targeting FoxM1/c-Myc pathway. *Oncotarget* **6**, 3988–4004 (2015). 25686834 10.18632/oncotarget.2905 PMC4414168

[43] Zhu, K. et al. Tumor exosomal miR-221-3p induces glycolysis through the LIFR/GLUT1 pathway to destroy the cerebral vascular endothelial cell barrier and promote breast cancer brain metastasis. *J. Transl. Med.* **23**, 1333 (2025). 41272827 10.1186/s12967-025-07372-8 PMC12639775

[44] Alanazi, A. et al. Identifying the roles of miR-17 in ciliogenesis and cell cycle. *Front. Cell Dev. Biol.* **12**, 1397931 (2024). 39268086 10.3389/fcell.2024.1397931 PMC11390542

[45] Alexandri, C. et al. MicroRNA profiling and identification of let-7a as a target to prevent chemotherapy-induced primordial follicles apoptosis in mouse ovaries. *Sci. Rep.* **9**, 9636 (2019). 31270341 10.1038/s41598-019-45642-w PMC6610114

[46] Long, X. B. et al. Let-7a microRNA functions as a potential tumor suppressor in human laryngeal cancer. *Oncol. Rep.* **22**, 1189–1195 (2009). 19787239 10.3892/or_00000554

[47] Saleh, A. D. et al. Cellular stress induced alterations in microRNA let-7a and let-7b expression are dependent on p53. *PLoS ONE* **6**, e24429 (2011). Erratum in: *PLoS ONE* **7** (2012). doi/10.1371/annotation/5c1dd48c-38e4-41ed-b827-748f62e28365; doi/10.1371/annotation/9b0f3eff-0376-43ca-b357-adfd8fc9ac7a. 22022355 10.1371/journal.pone.0024429 PMC3191136

